# HIV-1 Is a Poor Inducer of Innate Immune Responses

**DOI:** 10.1128/mBio.02834-18

**Published:** 2019-02-12

**Authors:** Oya Cingöz, Stephen P. Goff

**Affiliations:** a Department of Biochemistry and Molecular Biophysics, Columbia University Medical Center, New York, New York, USA; b Department of Microbiology and Immunology, Columbia University Medical Center, New York, New York, USA; c Howard Hughes Medical Institute, Columbia University Medical Center, New York, New York, USA; University of Michigan—Ann Arbor; Icahn School of Medicine at Mount Sinai; Boston University School of Medicine

**Keywords:** HIV-1, innate immunity, interferon-stimulated genes, interferons

## Abstract

Effective host immune responses against viral infection rely on the detection of the virus, activation of downstream signaling pathways, and the secretion of interferons (IFNs) and other cytokines. Many viruses can potently stimulate these responses, whereas the immune response against human immunodeficiency virus type 1 (HIV-1) remains relatively less well characterized. Here we show that HIV-1 infection with reporter viruses does not activate sensing pathways in cell lines and primary cells that are otherwise responsive to foreign nucleic acids. After entry into cells, reverse transcription and reporter expression occur without the virus ever being detected by cellular sensors or stimulating an interferon response. Using multiple methods, including the use of reporter cell lines for type I IFN and NF-κB pathway activation, quantifying mRNA levels for IFN-stimulated genes (ISGs), and assaying for markers of innate immune activation, we show that single-round pseudotyped HIV-1-based reporter viruses fail to induce innate immune responses.

## Abstract (precis)

Human immunodeficiency virus type 1 (HIV-1) continues to be a major burden to human health worldwide. How infected cells recognize and respond to HIV-1 infection is important in order to better understand the biology of the virus and the cellular pathways activated upon infection and to identify potential targets that interfere with viral replication. In this study, we investigated innate immune responses of different cell types following infection with single-cycle (replication-defective) HIV-1 reporter virus. We report that infection with a commonly used HIV-1 strain (lacking the *env*, *nef*, and *vpr* genes) does not measurably activate cellular defense mechanisms and that the virus is able to avoid recognition by cellular sensors.

## OBSERVATION

Innate immunity, a collection of responses activated upon identification of danger signals, provides immediate protection against pathogens. The magnitude of the response is carefully balanced to avoid damage to host tissues. As host responses can block their replication, many pathogens have evolved ways to either avoid detection or overcome innate immune pathways. Compared to many other virus families, retroviruses have been considered to be relatively poor inducers of innate immune responses, a view that has been challenged in recent years. Whether retroviruses stimulate an innate immune response in infected cells remains a topic under discussion, and there are various contexts in which human immunodeficiency type 1 (HIV-1) infection can stimulate type I interferon (IFN) production (reviewed in references 1 and 2).

In experiments with primary cells and cell lines, various steps in the viral replication cycle have been reported as being detected by host sensors (3–13). Infection has been suggested to trigger responses prior to reverse transcription, after reverse transcription, after nuclear entry, at the time of viral DNA integration, postintegration, and even in the absence of productive infection. These discrepancies likely reflect differences in cell type, virus strain, replication competence, mutations in the viral genome, the presence or absence of viral accessory genes, the envelope protein used for infection, and even the protocol used for cell differentiation. HIV-1 infection can often occur without robust induction of an innate immune response, in contrast to what occurs with many other viruses. Various manipulations, such as overexpression or depletion of factors in the host cell (14), introducing mutations into the viral genome (5, 6), and adding accessory genes from related retroviruses (9), can increase the host responses to infection.

The most important determinant that distinguishes transmitted founder (TF) viruses from those that arise during chronic infection is type I IFN resistance (15–17). As TF viruses are those that establish initial infection in the new host, there is reason to believe that IFN resistance is selected for and that host IFN responses propose a significant barrier to transmission. Further studies on how HIV-1 infection avoids or overcomes host interferon responses are therefore warranted. Here we examined the potential of an HIV-1 vector to induce innate immune activation in several commonly used cell types. Using pseudotyped reporter viruses, we show that infection with vesicular stomatitis virus glycoprotein (VSV-G)-pseudotyped, single-round HIV-1 reporter viruses do not measurably induce any of the markers of immune activation in several immunocompetent cells, despite successful infection. These data highlight the ability of HIV-1 and HIV-1-based vectors to evade detection in many settings.

**FIG 1:**
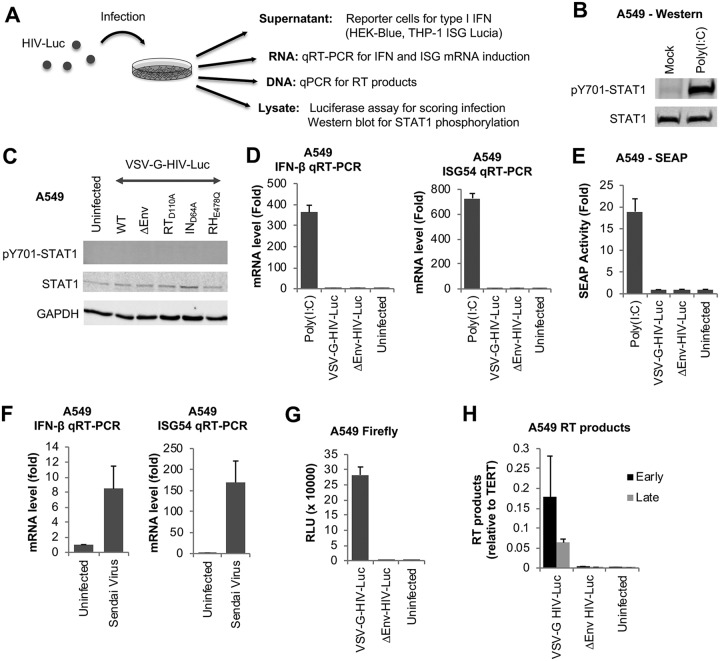
Single-round infection with HIV-1 reporter virus does not induce markers of innate immune activation in A549 lung epithelial cells. (A) Overview of experimental setup. (B) A549 cells were transfected with poly(I·C) at 4 µg/ml or mock transfected, and lysates were collected 4 h later and analyzed in a Western blot probed with the indicated antibodies. (C) Cells were uninfected or infected with VSV-G-pseudotyped, single-round HIV-Luciferase reporter (VSV-G-HIV-Luc), either wild-type (WT) or without an envelope (∆Env) or with the indicated mutations. Lysates were collected 1 day later and analyzed by Western blotting. (D) Cells were transfected with poly(I·C) at 4 µg/ml or infected with HIV-Luc with or without VSV-G Env. RNA was collected 4 h after transfection or 1 day after infection and analyzed by quantitative reverse transcription-PCR for IFN-β and ISG54 levels, normalized to actin. (E) Cells were treated as described for panel D, and culture supernatants at the same time points were collected and incubated with HEK Blue IFN-α/β reporter cells overnight. Secreted embryonic alkaline phosphatase (SEAP) activities in the supernatants were quantified 1 day later by absorbance measurement. (F) Cells were infected with Sendai virus, and RNA was isolated 1 day later and analyzed by quantitative reverse transcription-PCR. (G) Cells were infected with HIV-Luc with or without a VSV-G Env, and firefly luciferase activity was measured 2 days after infection. (H) Cells were infected as described for panel G, DNA was isolated 1 day later, and retroviral early and late RT products were quantified by qPCR. Data are averages of results from triplicates. Error bars denote standard errors of the means (SEM). Results from infections are normalized to results for uninfected cells, whereas results from transfections are normalized to results for mock-transfected controls. RLU, relative light units; TERT, telomerase reverse transcriptase.

We studied innate immune responses following HIV-1 infection in several immunocompetent cell types. We performed an array of experiments in which we scored cells for infection by reporter expression and quantification of reverse transcription (RT) products and assayed for markers of innate immune activation, such as IFN or IFN-stimulated gene (ISG) mRNA induction, type I IFN production, and STAT1 activation (Fig. 1A). A549 lung epithelial cells are responsive to foreign nucleic acids, as well as to infection with viruses causing an IFN response, such as Sendai virus (SeV). Indeed, transfection of poly(I·C) (4 µg/ml) induced a robust type I IFN response in these cells, evident by STAT1 phosphorylation 4 h after transfection (Fig. 1B). To assess potential innate immune responses to HIV-1 infection, we used the pNL4.3-Luc.E^–^R^–^ vector, which does not carry the *env*, *vpr*, or *nef* gene and expresses firefly luciferase upon infection. We transduced A549 cells with VSV-G-pseudotyped single-round reporter viruses (hereinafter HIV-Luc) and assayed for STAT1 phosphorylation 1 day after infection. Neither wild-type (WT) HIV-Luc nor viruses lacking reverse transcriptase (RT), integrase (IN), or RNase H (RH) activities induced STAT1 activation, demonstrating a lack of IFN signaling (Fig. 1C). We also quantified the mRNA levels of IFN-β and ISG54 (IFIT2) by quantitative reverse transcription-PCR. Within 4 h of transfection with 4 µg/ml poly(I·C), we detected a strong induction of both IFN-β and ISG54 mRNAs (Fig. 1D). Infection with HIV-Luc with or without VSV-G Env, however, failed to induce either mRNA 1 day after infection. We collected supernatants from infected cells and assayed for type I IFN release on HEK-Blue IFN-α/β reporter cells, which are stably transfected with a reporter construct consisting of multiple copies of an IFN-stimulated response element (ISRE) and an ISG54 minimal promoter that drive the expression of secreted embryonic alkaline phosphatase (SEAP). The supernatants from these cells can then be quantified for SEAP activity by a colorimetric assay, which is indicative of type I IFN in the sample. While supernatants from poly(I·C)-transfected cells induced strong SEAP expression, those from HIV-Luc-infected cells did not (Fig. 1E). As with poly(I·C) transfection, SeV infection successfully induced IFN-β and ISG54 induction in these cells (Fig. 1F).

To ensure that infection was successful, we measured firefly luciferase activity carried on the retroviral vector. There was a strong expression of luciferase in HIV-Luc-infected cells but not in cells infected with virus lacking an envelope (ΔEnv) (Fig. 1G). To demonstrate further that viral nucleic acids resulting from retroviral reverse transcription are present, we isolated DNA from infected cells 1 day after infection and measured early and late RT products by quantitative PCR (qPCR). We detected both early and late RT products in HIV-Luc-infected cells but not in cells infected with ΔEnv virus (Fig. 1H). In summary, we show that while VSV-G-pseudotyped single-cycle HIV-1 can infect cells efficiently, it evades innate immune recognition even in immunocompetent cells, such as A549 cells.

**FIG 2:**
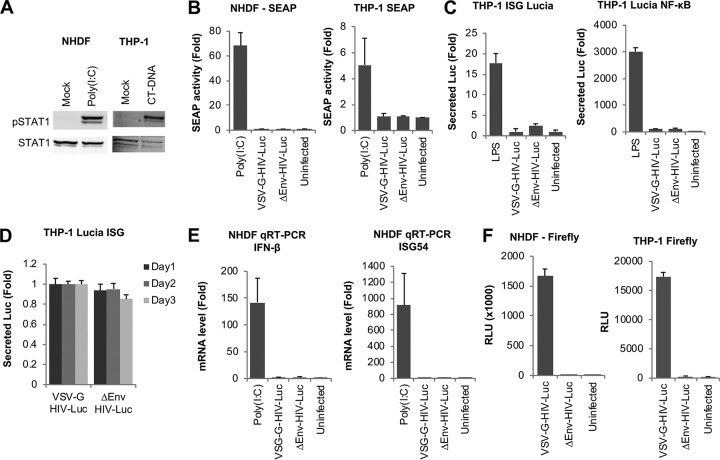
HIV-1 avoids innate immune recognition in a THP-1 monocyte cell line and NHDF primary fibroblasts. (A) NHDF and THP-1 cells were transfected with poly(I·C) or calf thymus DNA (CT-DNA) at 4 µg/ml or mock transfected, and lysates were collected 4 h later and analyzed in a Western blot probed with the indicated antibodies. (B) NHDF and THP-1 cells were transfected with poly(I·C) at 4 µg/ml or infected with HIV-Luc. Culture supernatants were collected 6 h after transfection or 1 day after infection and incubated with HEK Blue IFN-α/β reporter cells overnight. Secreted embryonic alkaline phosphatase (SEAP) activity was quantified 1 day later by absorbance measurement. (C) THP-1 Lucia ISG and THP-1 Lucia NF-κB reporter cells were treated with LPS or infected with WT or ΔEnv HIV-Luc. Supernatants were collected 1 day later and analyzed for secreted luciferase (Lucia) activity. (D) THP-1 Lucia ISG cells were infected with WT or ΔEnv HIV-Luc. Supernatants were collected at 1, 2, and 3 days after infection and assayed for secreted luciferase activity. (E) NHDF cells were transfected with poly(I·C) at 4 µg/ml or infected with HIV-Luc. RNA was collected 4 h after transfection or 1 day after infection and analyzed by quantitative reverse transcription-PCR for IFN-β and ISG54 levels. (F) NHDF and THP-1 cells were infected with HIV-Luc, and firefly luciferase activity was measured 2 days after infection. Data are averages of results from triplicates. Error bars denote SEM. Results of infections are normalized to results for uninfected cells, whereas results from transfections are normalized to results for mock-transfected controls.

We then studied innate immune responses against HIV-1 infection in two other cell types: the monocytic cell line THP-1 and primary fibroblasts (normal human dermal fibroblasts [NHDF]). We have shown previously that both cell types respond to foreign nucleic acids introduced by transfection and to infection with Sendai virus (18). Indeed, transfection of stimulatory nucleic acids, such as calf thymus DNA (CT-DNA) or poly(I·C) at 4 µg/ml, resulted in STAT1 phosphorylation, demonstrating the responsiveness of both cell types to foreign nucleic acids (Fig. 2A). To test for IFN induction upon infection of these cell types, we measured type I IFN production using the bioassay in HEK-Blue IFN-α/β reporter cells. As with the results obtained with A549 cells, supernatants from NHDF or THP-1 cells transfected with poly(I·C) resulted in high levels of SEAP activity, indicating type I IFN production, whereas cells infected with VSV-G-HIV-Luc failed to produce detectable levels of type I IFN (Fig. 2B).

We also used THP-1 Lucia (a type of luciferase) ISG and THP-1 Lucia NF-κB reporter cells, which express inducible secreted Lucia in response to type I IFN or NF-κB, respectively. Treatment of both cell types with lipopolysaccharide (LPS) resulted in substantial reporter expression, but infection with HIV-Luc did not (Fig. 2C). We observed similar results up to 3 days after infection (Fig. 2D). It is worth noting that Lucia and firefly luciferase enzymes do not cross-react and that they have distinct substrates.

The mRNAs for IFN-β and ISG54 were not induced by HIV-Luc infection of NHDF cells, where poly(I·C) transfection served as a positive control (Fig. 2E). We assayed for firefly luciferase activity on NHDF and THP-1 cells 2 days after infection, which yielded readily detectable reporter expression, indicating successful infection (Fig. 2F). Thus, the lack of innate immune activation by HIV-Luc in these cells is not due to a lack of infection or a lack of sensing pathway components but rather due to the virus not stimulating those pathways. In summary, we tested several different cell types that are highly responsive to foreign nucleic acids for their responsiveness to HIV-1 infection. Our data show that HIV-1 avoids innate immune sensing in infected cell lines and does not induce markers of innate immune activation under the conditions tested.

We find that infection with HIV-1-based viral vectors, in comparison to other viruses, are poor inducers of innate immune responses, a property that may have evolved under selective pressure of avoiding host resistance. The mechanism might involve the sequestering of viral nucleic acids; in particular, the HIV-1 capsid is thought to shield the incoming viral RNA and the reverse-transcribed viral DNA from cytoplasmic sensors (5). There is also evidence for the interaction of the viral capsid with specific host proteins preventing the recognition of viral infection (5, 6). In addition, several studies have suggested active inhibition of sensing pathways by HIV-1 accessory gene products (19–21). It should be noted that there are specific conditions under which HIV-1 can stimulate host innate immune pathways, depending on the infected cell type, replication competence of the virus, mutations in the viral capsid, and the presence of accessory genes, as well as the timing and scale of infection. The majority of studies that report the sensing of HIV-1 infection were performed with primary cells using replication-competent viruses (5, 6, 9, 10, 14, 22), although there are several reports of single-round replication-defective HIV-1 being sensed during reverse transcription (8, 9, 14).

In this study, three different cell types were used, all of which were validated for their responsiveness against foreign nucleic acids and Sendai virus infection (18). Despite efficient infection by a single-cycle HIV-1 strain, none of the cell types showed activation of innate immune markers; they showed neither type I IFN production nor ISG mRNA induction nor STAT1 phosphorylation for up to 3 days after infection. It is possible that increased production of viral nucleic acids and proteins such as those generated during spreading infection are required for efficient sensing in these cells. In spreading infection, nuclear export of intron-containing HIV-1 RNA can trigger type I IFN responses in a mitochondrial antiviral signaling (MAVS)-dependent manner (22). Primary lymphoid and myeloid cells may express crucial factors necessary for HIV-1 sensing, which might have been lost in the cell types used in our system. Importantly, bypassing HIV-1 Env-mediated entry into cells by VSV-G pseudotyping might interfere with the recognition pathway. In addition, lab-adapted strains of HIV-1 are likely to have incurred mutations that render them less immunogenic than clinical isolates or transmitted founder (TF) viruses. Notably, a determining characteristic of TF viruses is their relative resistance to IFN (17), suggesting that innate immune responses during transmission *in vivo* form enough pressure for TF viruses to be selected against IFN sensitivity.

Myeloid cells are infected poorly with HIV-1 due to low triphosphate levels and the role of SAMHD1 in inhibiting reverse transcription (23, 24). This block can be overcome by expressing Vpx from HIV-2 or some simian immunodeficiency viruses (SIVs), which target SAMHD1 for degradation. The results of innate immune activation observed in primary cells upon infection with Vpx-containing viruses should be interpreted with caution, as Vpx itself can potentially cause the activation of innate immune responses due to SAMHD1 degradation, regardless of HIV-1 infection. A similar argument might be made for TREX1-depleted cells, as mutations in both genes are associated with the autoimmune disease Aicardi-Goutières syndrome (AGS). In their absence, increased IFN responses due to the activation of sensors by endogenous nucleic acid ligands ensue. It is therefore important to tease apart the potential of HIV-1 to stimulate cellular sensors and induce the production of cytokines in the absence of such manipulations.

### Cells and viruses.

293T, NHDF, and A549 cells (ATCC) were grown in Dulbecco’s modified Eagle’s medium (DMEM) supplemented with 10% fetal bovine serum (FBS) plus 1× penicillin-streptomycin, and THP-1 cells (ATCC) were grown in RPMI 1640 with the same supplements. THP-1 Lucia ISG and THP-1 Lucia NF-κB cells (InvivoGen) were grown in RPMI 1640 with 10% FBS, 1× penicillin-streptomycin, and 100 µg/ml phleomycin D1 (Zeocin). Viruses were produced by transfection of 293T cells using Lipofectamine 2000 (Invitrogen) with the pNL4.3.E^–^R^–^ Luc vector and a VSV-G plasmid or, in the case of ΔEnv virus, with an empty vector plasmid instead of VSV-G. Viruses were collected 2 days posttransfection, and their titers were determined in 293T cells. Virus yields were analyzed by p24 enzyme-linked immunosorbent assay (ELISA; ABL) or by Western blotting. Cells were infected with up to 220 ng/ml p24-containing viruses.

### Nucleic acids and antibodies.

RNA and DNA were isolated using Qiagen RNeasy and DNeasy kits. RNA was DNase treated with a Turbo RNase-free DNase kit (Ambion), and DNase was inactivated, cDNA was synthesized (ABI cDNA synthesis kit), and qPCR was performed using TaqMan Universal 2× master mix (Roche). TaqMan primer-probe sets were purchased from Thermo Scientific (IFNB1, Hs01077958_s1; CXCL10, Hs00171042_m1; ISG54, Hs00533665_m1; ACTB, Hs99999903_m1). CT-DNA and poly(I·C) were from Sigma, LPS was from Santa Cruz Biotechnology, and antibodies were as follows: pY701-STAT1 and STAT1 (Cell Signaling Technology), anti-glyceraldehyde-3-phosphate dehydrogenase (VWR), and anti-p24 (Abcam).

### Reporter assays.

For Firefly luciferase assays, cells were lysed 2 days after HIV-Luc infection and quantified by luciferase assay (Promega). For Lucia assays, supernatants from THP-1 Lucia cells were collected after 1 to 3 days of treatment or infection and quantified with Quanti-Luc reagent (InvivoGen). For SEAP assays, supernatants from infected or transfected cells were collected 1 day after infection and incubated with HEK-Blue IFN-α/β reporter cells overnight, and SEAP activity was quantified the next day using Quanti-Blue reagent (InvivoGen) by absorbance measurement at 260 nm. All measurements were performed with an Omega POLARstar plate reader (BMG Labtech).
